# Circular RNA-based HPV16 therapeutic vaccine elicits potent and durable antitumor immunity

**DOI:** 10.1186/s13046-026-03640-7

**Published:** 2026-01-07

**Authors:** Rong Zhou, Chonghui Li, Kunlun Xiang, Lifang Cui, Yin Rong, Leshi Li, Minliang Zhu, Jing Zeng, Lu Gao

**Affiliations:** 1 Therorna Inc., Beijing, China; 2 Therorna Shanghai Co., Ltd., Shanghai, China

**Keywords:** Human papillomavirus 16, Therapeutic vaccine, Circular RNA, Lipid nanoparticles, RNA sequencing, Tumor microenvironment, PD-L1

## Abstract

**Background:**

Human papillomavirus (HPV) 16 infection is associated with several human malignancies. Developing therapeutic vaccines holds great potential for patients who do not benefit from standard care. Circular RNA (circRNA) is an emerging next-generation platform for cancer vaccine development owing to its superior stability and convenient manufacturing process. Herein, we report development of a synthetic circRNA encoding fused HPV16 E7/E6 antigens encapsulated with lipid nanoparticles (LNP) to treat HPV16-related solid tumors.

**Methods:**

The immunogenicity and anti-tumor immune response of the LNP-circRNA vaccine was determined in naïve C57BL/6 mice and TC-1 tumor-bearing mice, respectively. Changes in immune cells were examined using flow cytometry and immunofluorescence assay. RNA sequencing was used to identify differentially expressed genes and changes in the tumor microenvironment (TME) of tumors treated with LNP-circRNA^E7E6^ and empty LNP. Anti-tumor efficacy was further evaluated in LNP-circRNA^E7E6^ vaccine combined with anti-PD-L1 antibody treatment.

**Results:**

Prime-boost vaccination with LNP-circRNA^E7E6^ induced a large pool of functional antigen-specific cytotoxic T cells in both the peripheral blood and spleen. This immunization led to profound changes in the TME, characterized by the upregulation of immune activation genes, heavy infiltration of immune cells, and polarization toward a proinflammatory state. Consequently, circRNA^E7E6^ immunization could mediate complete tumor regression and prevent tumor growth. Moreover, vaccination sensitized non-inflamed tumors to immune checkpoint blockade therapy.

**Conclusions:**

The present study results demonstrate that LNP-circRNA^E7E6^ vaccine is capable of eliciting robust anti-tumor immunity in the periphery and TME, highlighting the potential for treating HPV16-related cancers and preventing tumor recurrence.

**Supplementary Information:**

The online version contains supplementary material available at 10.1186/s13046-026-03640-7.

## Background

Persistent human papillomavirus (HPV) infection is thought to be responsible for 90% of cervical cancers and 70% of oropharyngeal head and neck cancers [1]. Among the more than 200 HPV types, HPV16 is particularly prevalent. HPV16 has been detected in 52.9% of HPV-associated cervical cancers and 91.2% of head and neck cancers [2]. Current standard treatments for HPV-associated malignancies, including surgery, chemotherapy, and radiotherapy, offer limited benefits to patients with recurrent or relapsed tumors [3, 4]. Although prophylactic HPV vaccines have been approved to prevent new infections, they lack therapeutic efficacy in individuals with pre-existing infections or established neoplasia [5–8]. Hence, there is an urgent need to develop effective immunotherapies, such as therapeutic cancer vaccines, against HPV16-associated malignancies.

It has been demonstrated that HPV can promote tumorigenesis using E6 and E7 oncoproteins and by inhibiting two crucial tumor suppressors, p53 and pRb [9–11], respectively. This oncogenic activity, combined with several key characteristics, makes E6 and E7 ideal therapeutic vaccine targets. First, these oncoproteins are abundantly and specifically expressed in precancerous and cancerous lesions, minimizing off-target risks. Second, E6 and E7 are essential for oncogenic transformation, which will reduce the likelihood of immune escape via antigen loss. The immunologic responses against these proteins have been well characterized in both preclinical and clinical studies [12, 13], providing compelling evidence regarding their therapeutic vaccine potential.

The efficacy of a therapeutic cancer vaccine is critically dependent on the cellular immune response, specifically the induction of robust antigen-specific CD8^+^ T-cell responses via effective major histocompatibility class I (MHC-I) antigen presentation [14]. In the classical pathway, cytosolic antigens within an antigen-presenting cell are degraded by the proteasome. The resulting peptides are transported into the endoplasmic reticulum (ER) by the transporter associated with antigen processing (TAP), where they are loaded onto MHC-I molecules. This critical assembly process is orchestrated by a multi-protein peptide-loading complex comprising TAP, tapasin, calreticulin (CRT), and ERp57, which ensures the proper assembly of peptide–MHC complexes and subsequent release from the ER [15]. Leveraging this mechanism, one key strategy in therapeutic vaccine design involves fusing the target antigen with specific sequences or domains to facilitate its processing and loading onto MHC-I molecules. The fusion of ER transport and retention signals of CRT to HPV16 E6 and E7 in a DNA vaccine exemplifies this approach [16]. As a crucial chaperone in the peptide-loading complex, CRT promotes the association of antigenic peptides with MHC-I molecules, ultimately leading to more effective T-cell activation.

Herein, we describe the development a novel circular RNA vaccine that encodes HPV16 oncoproteins E6 and E7, formulated in lipid nanoparticles (LNP) for intramuscular (i.m.) administration. Circular RNA^E7E6^ (circRNA^E7E6^) encoded a fusion protein containing a leading signal peptide of CRT protein, mutated E7/E6 sequence of HPV16, immune modulation helper peptide (P2P16) derived from tetanus toxoid, and C-terminal ER retention peptide (KDEL). Our results demonstrated that immunization with circRNA^E7E6^ resulted in complete remission of HPV16-associated TC-1 tumors and significantly prolonged survival in tumor-bearing mice. The present results demonstrate the potent therapeutic efficacy of circRNA^E7E6^ and support further clinical investigation as a potential treatment for HPV16-positive recurrent or metastatic cancers.

## Methods

### circRNA^E7E6^ vaccine preparation and cell lines

We used a split internal ribosomal entry site (IRES) strategy to produce circular RNAs using T4 RNA ligase 2, as previously described [17]. Briefly, the split site was rationally designed in a coxsackievirus B3 (CVB3) IRES, and the open reading frame sequence was flanked by the two split parts of the IRES at the 5' and 3' ends. Rather than introduce an exogenous splint, the complementary paring sequence of the split IRES was taken as a splint to generate the nick serving as the catalytic substrate of T4 RNA ligase. circRNAs were produced via in vitro transcription using T7 RNA polymerase. The DNA template was then removed via DNase I treatment, and the linear RNA precursors were ligated using T4 RNA ligase 2. Finally, the ligated circRNAs were enriched with RNase R and purified using a column. Subsequently, the purified RNA molecules were analyzed in denaturing polyacrylamide gel. For LNP formulation, purified circRNA in Tris–HCl buffer (pH 4.0) was mixed with an ethanolic solution of lipids (SM-102, DSPC, cholesterol, and PEG-DMG at a 50:10:38.5:1.5 molar ratio) using a NanoAssemblr microfluidic system at a 3:1 ethanol-to-aqueous volume ratio. The resulting LNP-circRNA was then buffer-exchanged into a sucrose-containing Tris–HCl buffer, concentrated using ultrafiltration, sterile-filtered (0.22 µm), and stored at − 80°C. The particle size distributions of the LNP were determined by dynamic light scattering (DLS) using a Zetasizer Pro instrument (Malvern Panalytical, UK). Encapsulated RNA was subsequently quantified using a Quant-IT® Ribogreen Assay Kit (Catalog No: R11490, Invitrogen) following the manufacturer’s instructions. The murine TC-1 cell line, which express the HPV16 oncogenes E6 and E7 as well as activated human *HRAS* oncogenes, were used to establish a preclinical tumor model of HPV16-driven malignancy [18]. TC-1 cells were grown in RPMI 1640 supplemented with 100 U/mL of penicillin and 100 µg/mL of streptomycin and 10% fetal bovine serum (FBS). HEK293T cells were maintained in Dulbecco’s Modified Eagle Medium supplemented with 100 U/mL of penicillin and 100 µg/mL of streptomycin and 10% FBS.

### Western blot

Total protein from LNP-transfected cell lysates was quantified using a BCA Protein Assay Kit (Catalog No: P0012, Beyotime). For deglycosylation, lysates were treated with PNGase F (Catalog No: P0704S, NEB) at 37 °C for 1 h. Aliquots containing 10 µg of total protein were resolved by 10% SDS-PAGE and transferred to PVDF membranes using the Trans-Blot Turbo Transfer System (Bio-Rad). Membranes were blocked for 1 h using 5% non-fat dry milk in PBST (0.1% Tween-20 in PBS), followed by overnight incubation at 4 °C with primary antibodies against HPV16 E7 (Catalog No: sc-6981, Santa Cruz), HPV16 E6 (Catalog No: IG-E6-6F4, Euromedex), or Tubulin (Catalog No: AF2835, Beyotime). Subsequently, the membranes were incubated with an HRP-conjugated secondary antibody for 2 h. Chemiluminescent signals were detected using an NcmECL Ultra substrate (Catalog No: P10100, NCM) and captured with the ChemiDoc Imaging System (Bio-Rad). The bands were analyzed using ImageJ software (National Institutes of Health, Bethesda, MD, USA).

### Immunofluorescence

HEK293T cells were seeded at a density of 4 × 10^5^ cells/well onto glass coverslips in 12-well plates. The cells were transfected with 3 µg of the indicated LNP-encapsulated circRNAs. At 24 h post-transfection, cells were washed with PBS, fixed with 4% paraformaldehyde for 10 min, and permeabilized with 0.25% Triton X-100 for 15 min. Following blocking with 2% BSA for 40 min, the cells were incubated overnight at 4 °C with primary antibodies: mouse anti-HPV16 E7 (1:100, Catalog No: sc-6981, Santa Cruz) and rabbit anti-calnexin (1:200, Catalog No: 10427-2-AP, Proteintech). Subsequently, the cells were incubated for 1 h with goat anti-mouse IgG (H + L) Alexa Fluor 488 (1:1000, Catalog No: P0179, Beyotime) and goat anti-rabbit IgG (H + L) Alexa Fluor 555 (1:1000, Catalog No: C1002, Beyotime). Coverslips were then mounted using Antifade Mounting Medium with DAPI (Catalog No: P0131, Beyotime). Images were acquired on a Nikon A1RSi + confocal microscope using a 100 ×/1.45 NA oil-immersion objective and NIS-Elements software (Nikon, Tokyo, Japan). The images were analyzed using ImageJ software (National Institutes of Health, Bethesda, MD, USA).

### Animal studies

We obtained 6- to 8-week-old female C57BL/6 mice from Shanghai Lingchang Biotechnology Co., Ltd. The mice were maintained under specific pathogen-free conditions at the Shanghai Model Organisms Center (SMOC). All experimental procedures were approved by the Institutional Animal Care and Use Committee of SMOC. To establish a TC-1 syngeneic tumor model, 1.0 or 2.5 × 10^5^ TC-1 cells were injected subcutaneously (s.c.) into the right flank of each mouse. When tumors reached a volume of 40–150 mm^3^, the mice received a 50 µL intramuscular injection of LNP-encapsulated circRNA^E7E6^ vaccine or vehicle into the gastrocnemius muscle of the hind limb. Dosages were varied by adjusting the concentration within this fixed volume. In some experiments, anti-PD-L1 antibody atezolizumab or human IgG1 isotype control antibody produced by Biointron (Taizhou, China) was given intraperitoneally at 200 μg/mouse every 3 to 4 days. The animals were euthanized when predefined endpoints were reached or the average tumor volume exceeded 2000 mm^3^. Tumor volumes were calculated using caliper measurements according to the formula V = (L × W^2^)/2, where V is the tumor volume, L is tumor length, and W is tumor width. The relative tumor volume (RTV) was calculated as: 100% × (tumor volume on day X)/(tumor volume on day 0). The tumor growth inhibition (TGI) rate was calculated as follows: (1 – [RTV of treated group]/[RTV of control group]) × 100%.

### Tumor tissue preparation

The mice were euthanized with CO_2_, and then tumor tissues were collected, cut into small pieces, and digested in 2 mg/mL collagenase type IV (Catalog No: 17104019, Gibco) at 37 °C for 1 h. Single cell suspensions of tumor tissues were prepared in PBS containing 10% FBS and 2 mM EDTA. Tissues were disrupted on the surface of a 70-µm filter using the plunger of a 1-mL syringe.

### RNA sequencing (RNA-seq) and data analysis

TC-1 tumors were surgically isolated from C57BL/6 tumor-bearing mice and cryopreserved in liquid nitrogen. RNA extraction, messenger RNA (mRNA) library construction, and sequencing were conducted by Azenta Life Sciences (Suzhou, China). For RNA-seq data analysis, raw sequencing reads were checked for quality using FastQC (v.0.10.1, https://www.bioinformatics.babraham.ac.uk/projects/fastqc/) and processed using Cutadapt (v.1.9.1) to remove adapters and poor-quality bases. Reads shorter than 75 bp were also discarded [19]. Clean reads were aligned to the mm10 mouse reference genome using HISAT2 (v.2.2.1) [20, 21], and gene-level counts were quantified with HTSeq (v.0.6.1) [22]. Differential expression analysis was performed using the DESeq2 (v.1.34.0) R package [21]. FPKM (fragments per kilobase million) was calculated and used for gene expression plot generation. Genes differentially expressed at least two-fold were used for further analysis (Gene Ontology [GO], gene set enrichment analysis [GSEA], and ImmuCellAI-mouse inference). RNA-seq data in this study have been deposited at Gene Expression Omnibus (http://www.ncbi.nlm.nih.gov/gen/) under accession number GSE300736.

### Tetramer staining

To assess antigen-specific CD8^+^ T cell responses, splenocytes from immunized mice were isolated and cultured overnight in 96-well plates (3 × 10^6^ cells/well) with a 2 µg/mL HPV16 E6/E7 peptide pool. Following stimulation, cells were harvested and stained with Fixable Viability Stain 510 (Catalog No: 564406, BD) and an anti-mouse CD16/CD32 antibody (Catalog No: 553142, BD) for Fc blocking. Cells were then incubated with E7 tetramer (H-2Db HPV16 E7 Tetramer-RAHYNIVTF-APC; Catalog No: TB-5008-02, MBL Life Science) at 4 °C for 1 h. Subsequently, surface staining was performed by incubating cells with APC/Cyanine7-conjugated anti-mouse CD45 (Catalog No: 103116, Biolegend), FITC-conjugated anti-mouse CD8 antibodies (Catalog No: 553030, BD) and PE-conjugated anti-mouse CD3 antibodies (Catalog No: 553064, BD) for 30 min at 4°C. After washing them with DPBS, the stained cells were acquired on a FACSCalibur™ flow cytometer. In some experiments, peripheral blood was isolated directly for tetramer staining without peptide pool stimulation. Data was analyzed using FlowJo 10.9.0 software (BD Biosciences).

### Interferon gamma (IFN-γ) enzyme-linked immunosorbent spot (ELISpot) assay

A single cell suspension of mouse spleen tissues was collected at the endpoint of the animal studies. ELISpot assay was performed to evaluate the frequency of background and antigen-stimulated spot-forming units, following the protocol of the Mouse IFN-γ ELISpot PLUS (ALP) Kit (Catalog No: 3321-4AST-2, Mabtech). Briefly, 1 × 10^5^ of freshly isolated splenocytes per well were stimulated with 10 ng/mL phorbol 12-myrstate 13-acetate and 0.5 μg/mL ionomycin as positive control wells, media alone as negative control wells, or 2.0 μg/mL HPV16 E6/E7 peptide pool (Genscript, Nanjing, China) as experimental wells. In some experiments, the P2P16 peptide pool (Genscript, Nanjing, China) was used as a stimulus to assess TT epitope–specific T-cell activity. For certain assays, CD4^+^ T cells were enriched from the splenocyte population via magnetic depletion of CD8^+^ cells (Catalog No: 11462D, Invitrogen). Plates were read on an AID iSpot ELISpot reader and analyzed using AID ELISpot Software (AID Autoimmun Diagnostika GmbH, Strassberg, Germany).

### Flow cytometry analysis of peripheral blood and tumor-infiltrating cells (TILs)

Whole blood or single cell suspensions of tumor tissues were stained with fluorescent dye-conjugated antibodies. The antibodies and reagents used were as follows: CD3 (Catalog No: 100220, Biolegend, San Diego, CA, USA), CD4 (Catalog No: 563151, BD Biosciences, San Jose, CA, USA), CD8 (Catalog No: 100742, Biolegend), CD44 (Catalog No: 103049, Biolegend), CD45 (Catalog No: 103116, Biolegend), CD62L (Catalog No: 104428, Biolegend), E7 tetramer (H-2Db HPV16 E7 Tetramer-RAHYNIVTF-APC; Catalog No: TB-5008-02, MBL Life Science), IFN-γ (Catalog No: 505830, Biolegend), perforin (Catalog No: 154306, Biolegend), granzyme B (GzmB; Catalog No: 515403, Biolegend), PD-1 (Catalog No: 135225, Biolegend), and fixable viability dye (Catalog No: 423101, Biolegend). All experiments were conducted on FACSverse (BD Biosciences, Piscataway, NJ, USA) and CytoFLEX (Beckman Coulter, Indianapolis, IN, USA) flow cytometers and analyzed using FlowJo 10.9.0 software (BD Biosciences).

### Immunofluorescence analysis of TILs

Tumor tissues were embedded in paraffin after being fixed in 4% paraformaldehyde for more than 48 h. Then, 4-μm sections were cut from FFPE tumor blocks. Tumor sections were deparaffinized and underwent heat-induced antigen retrieval using AR9 buffer. Samples were stained with anti-CD8 alpha antibody (ERP21769, Abcam). Visualization was performed with a Novo-light TSA 4-Color IHC Kit (Shanghai WiSee Biotechnology Co., Ltd.). Images were acquired on a Pannoramic MIDI II Digital Scanner (3D HISTECH, Budapest, Hungary).

### Statistical analysis

Data were analyzed using GraphPad Prism 10 statistical software (San Diego, CA, USA). Experimental values are expressed as means ± standard deviations (SDs), unless otherwise indicated. For comparisons between two groups, we used an unpaired Student’s *t*-test. For comparisons of more than two groups, we used one-way analysis of variance (ANOVA). We considered *P* values < 0.05 to indicate statistical significance.

## Results

### Design of the HPV LNP-circRNA vaccine

In this study, a therapeutic circRNA vaccine for HPV16-related cancer was designed to elicit specific and robust anti-HPV T-cell responses. circRNA^E7E6^ encoded the non-oncogenic HPV16 E6 and E7 mutants, which were fused in an E7-E6 orientation. To eliminate oncogenicity, the binding motifs of E6 and E7 to p53 and pRb were mutated or deleted [23, 24]. To further enhance immunogenicity, the antigen was engineered to include an N-terminal calreticulin (CRT) signal peptide and a C-terminal KDEL retention sequence for endoplasmic reticulum (ER) targeting, along with the P2 and P16 helper epitopes from tetanus toxoid (TT) for the provision of CD4^+^ T-cell help [25]. As a reference control, we also generated constructs encoding the antigen with only the ER-targeting sequences (circRNA^E7E6−ER^) or only the TT helper epitopes (circRNA^E7E6−TT^), alongside a non-engineered version lacking both modifications (circRNA^∆E7E6^) for comparison.

**Fig. 1:**
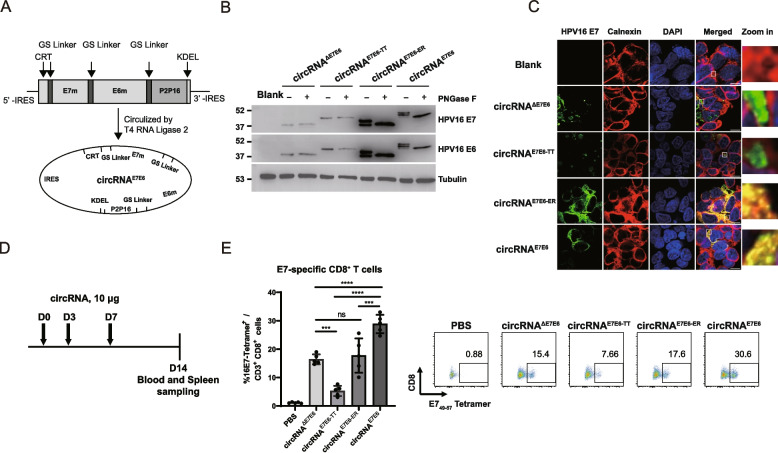
Design, characterization, and superior in vivo immunogenicity of the circRNA^E7E6^ vaccine. **A** Schematic diagram of the circRNA^E7E6^ construct design. CRT, human calreticulin signal peptide; E7m/E6m, mutated HPV16 antigens; P2P16, tetanus toxoid CD4^+^ epitopes; KDEL, ER retention signal. **B** Western blot for antigen expression in transfected HEK293T cells. Lysates were treated with (+) or without (−) PNGase F to assess glycosylation. Tubulin served as a loading control. **C** Confocal microscopy showing subcellular localization of indicated antigens (green) and the ER marker calnexin (red) in transfected HEK293T cells. Nuclei were stained with DAPI (blue). Co-localization is shown in yellow. Scale bar, 10 μm. **D** Schematic diagram of the immunization schedule. C57BL/6 mice were injected i.m. with 10 μg of LNP-circRNA on days 0, 3, and 7, with analysis on day 14. **E** Quantification of circulating HPV16 E7 tetramer-positive CD8^+^ T cells via flow cytometry. Representative pseudocolor plots are shown. Data in (**E**) are shown as means ± SDs. Significance was analyzed using one-way ANOVA with multiple comparisons. ** *P* < 0.01, *** *P* < 0.001, ****P < 0.0001

As described in the Methods section, RNA was circularized using a split IRES strategy with T4 RNA ligase 2, resulting in the final circRNA products, where a full-length CVB3 IRES element was positioned upstream of the antigen-coding sequence to initiate translation (Fig. 1A). Following efficient circularization (Supplementary Fig. S1A), circRNA was encapsulated into an LNP-based delivery system. The encapsulation efficiency was above 90%, with an average diameter of approximately 90 nm (Supplementary Fig. S1B). Western blot analysis showed the antigens were successfully expressed. Further characterization revealed that the engineered E7E6 and E7E6-ER antigens were N-linked glycoproteins. They migrated as distinct bands that collapsed into a single, faster-migrating band after endoglycosidase PNGase F treatment. In contrast, control antigens (circRNA^∆E7E6^ and circRNA^E7E6−TT^) consistently showed a single, PNGase F-insensitive band (Fig. 1B). Confocal microscopy further confirmed the subcellular localization of the antigens. The engineered E7E6 and E7E6-ER antigen largely co-localized with the ER marker calnexin, indicating predominant ER localization. In contrast, the unmodified and circRNA^E7E6−TT^ antigens displayed a diffuse cytoplasmic and nuclear distribution with punctate aggregates (Fig. 1C). These localization differences may underline both the distinct glycosylation profiles, as N-linked glycosylation occurs co-translationally in the ER [26], and the markedly increased protein expression, which may be attributable to enhanced stability within the ER [27].

To assess the in vivo immunogenicity of the circRNA constructs, we immunized C57BL/6 mice with 10 µg of each vaccine in a three-dose regimen. Seven days after the last dose (day 14), blood and spleen samples were collected for subsequent analyses (Fig. 1D). Tetramer staining with H2-Db MHC I tetramers carrying the E7_49_–_57_ epitope revealed that ER-targeting alone offered only modest improvement, and P2P16 alone even diminished the responses (Fig. 1E). This suppressive effect likely due to immunodominant competition for MHC-I presentation, where peptides from the potent helper sequence outcompete the tumor antigen for MHC class I presentation, a known challenge in vaccine design [28, 29]. Crucially, our inclusion of an ER-targeting signal was specifically designed to bias antigen processing toward MHC I presentation and enhance CD8^+^ T activation. This strategy proved highly effective: the circRNA^E7E6^ construct, which integrates both elements, significantly enhanced the E7-specific CD8^+^ T-cell response (Fig. 1E). This enhanced activation was also complemented by robust T-helper activity, evidenced by strong P2P16-specific IFN-γ release from CD4^+^ T cells (Supplementary Fig. S2). Collectively, the combination of the ER-targeting sequence with the TT epitope resulted in significantly greater antigen-specific T-cell responses compared to the other three constructs, highlight circRNAE7E6 as the most promising vaccine candidate.

### circRNA^E7E6^ vaccination induces antigen-specific CD8^+^ T-cell responses in mice

**Fig. 2:**
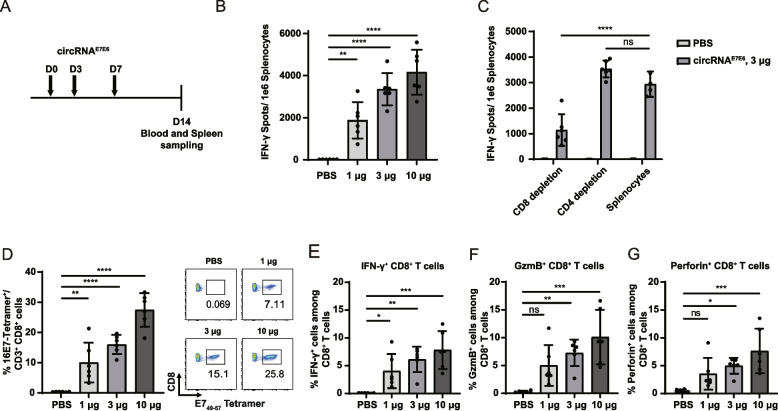
Immunogenicity of the circRNA^E7E6^ vaccine. **A** Schematic diagram of the vaccination procedure. Mice were immunized with different doses of circRNA^E7E6^ vaccine (1, 3, and 10 μg) on days 0, 3 and 7. Mice were killed 1 week after the final dose (day 14). **B** IFN-γ-secreting splenocytes were identified using ELISpot assay. **C** CD4 versus CD8 depletion analysis revealed the immune response phenotype. IFN-γ-secreting splenocytes were detected using ELISpot assay. **D** The percentage of HPV16 E7 tetramer-positive CD8^+^ T cells was determined via flow cytometric analysis. In splenocytes, the percentages of antigen specific IFN-γ-secreting (**E**), granzyme B-secreting (**F**), and perforin-secreting (**G**) CD8^+^ T cells were determined via flow cytometric analysis. Data are shown as means ± SDs. Significance was analyzed using one-way ANOVA with multiple comparisons (**B**–**G**). * *P* < 0.05, ** *P* < 0.01, *** *P* < 0.001, **** *P* < 0.0001, ns, not significant

To comprehensively evaluate the immunogenicity of circRNA^E7E6^ in vivo, C57BL/6 mice (*n* = 6 in each group) were injected i.m. with three different doses (1 µg, 3 µg, or 10 µg) of LNP-circRNA^E7E6^ on a schedule (days 0, 3, and 7), a control group received PBS only. Peripheral blood and splenocytes were harvested 1 week after the final immunization (Fig. 2A). Antigen-specific IFN-γ secretion in response to HPV16 E6/E7 peptide pool stimulation was quantified using ELISpot assay. Immunization with circRNA^E7E6^ induced a significant, dose-dependent increase in IFN-γ-secreting splenocytes compared with the control group (Fig. 2B). Depletion of CD8^+^ T cells, but not CD4^+^ cells, dramatically reduced IFN-γ spot formation, confirming that IFN-γ production induced by circRNA^E7E6^ is mediated mainly by CD8^+^ T cells (Fig. 2C). Furthermore, tetramer staining revealed markedly higher frequencies of E7-specific CD8^+^ T cells in the peripheral blood of all vaccinated groups, as compared with PBS controls. This response was dose-dependent, with E7-specific CD8^+^ T-cell proportions reaching 10%, 16%, and 27% following immunization with 1 µg, 3 µg, and 10 µg of circRNA^E7E6^, respectively (Fig. 2D and Supplementary Fig. S3). Consistently, splenic CD8^+^ T cells from mice immunized with circRNA^E7E6^ displayed dose-dependent increases in IFN-γ, GzmB, and perforin production (Fig. 2E–G and Supplementary Fig. S4). These results demonstrated that circRNA^E7E6^ could elicit robust, dose-dependence, and antigen-specific immune responses against HPV16.

### circRNA^E7E6^ vaccination effectively inhibits tumor progression

**Fig. 3:**
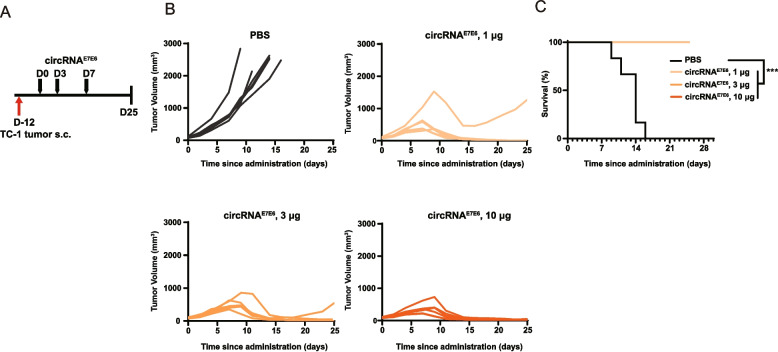
Therapeutic efficacy of circRNA^E7E6^ vaccine in the TC-1 syngeneic tumor model. **A** Schematic diagram of the vaccination procedure. C57BL/6 mice were injected s.c. with 1.0 × 10^5^ TC-1 cells in the right flank. After 12 days, tumor volumes reached an appropriate level (100 mm^3^). Tumor-bearing mice were immunized i.m. three times (days 0, 3, and 7) with circRNA^E7E6^ vaccine (1, 3, 10 μg) or PBS control. **B** Kinetics of TC-1 tumor growth and **C** overall survival are shown. Tumor volumes were measured three times a week during the study. Tumor growth curves are shown for individual animals. Survival curves presented as Kaplan–Meier curves. *** *P* < 0.001 when comparing vaccination groups with the control group using a log-rank test

Given the promising results obtained from the immunogenicity study, we investigated the anti-tumor efficacy in vivo of circRNA^E7E6^ using a TC-1 syngeneic tumor model. Treatment was initiated when tumors reached an average volume of 100 mm^3^. Mice received i.m. injections of circRNA^E7E6^ at doses of 1, 3, and 10 µg/mouse (Fig. 3A).

Immunization of circRNA^E7E6^ was associated with a rapid tumor regression after the first week of treatment initiation (Table 1). In contrast, mice in the PBS control group experienced progressive tumor growth and had to be euthanized from day 9 owing to a high tumor burden. At that time, the tumor growth inhibition (TGI) rates reached 83.36%, 85.87% and 92.45% in groups injected with 1, 3, and 10 µg of circRNA^E7E6^, respectively (Table 1). By day 16, all mice in the control group had been humanely euthanized. In contrast, no mice in the treatment group met the criteria for euthanasia, and most were tumor-free by the end of the study (day 25), significantly increasing each animal’s life span (Fig. 3B and C). These results indicated that circRNA^E7E6^ effectively inhibited TC-1 tumor growth and offered particularly improved survival benefits.

**Table 1:** Tumor growth inhibition (TGI) rates from days 0 to day 9

Group		PBS	circRNA^E7E6^ 1 μg	circRNA^E7E6^ 3 μg	circRNA^E7E6^ 10 μg
**N**^**#**^		**6**	**6**	**6**	**6**
Day 0	Tumor volume (mm^3^)	95.16 ± 5.91	95.31 ± 5.72	95.19 ± 5.31	95.46 ± 5.4
	TGI (%)	-	-	-	-
Day 2	Tumor volume (mm^3^)	222.70 ± 36.28	170.85 ± 21.91	154.49 ± 13.89	144.56 ± 13.64
	TGI (%)	-	23.41	30.65	35.30
Day 4	Tumor volume (mm^3^)	442.71 ± 50.14	332.75 ± 31.94	284.42 ± 20.92	256.51 ± 30.19
	TGI (%)	-	24.96	35.78	42.24
Day 7	Tumor volume (mm^3^)	851.14 ± 128.93	554.43 ± 109.78	477.97 ± 44.18^*^	353.66 ± 69.92^**^
	TGI (%)	-	34.96	43.87	58.58
^†^Day 9	Tumor volume (mm^3^)	1462.41 ± 277.21	511.41 ± 205.81^****^	498.35 ± 83.91^****^	345.16 ± 91.38^****^
	TGI (%)	-	65.08	65.93	76.47

Data are presented as means ± SEMs, Statistical significance was analyzed using one-way ANOVA with multiple comparisons between the PBS control and circRNA^E7E6^ treatment groups

N^#^: number of mice per group

^**†**^Day 9: The day when mice in the control group began to reach the humane endpoint owing to a high tumor burden

^*^P < 0.05, ^**^P < 0.01, ^***^P < 0.001, ^****^P < 0.0001

### circRNA^E7E6^ vaccination promotes strong infiltration of antigen-specific T cells into the tumor site and memory CD8^+^ T-cell induction

**Fig. 4:**
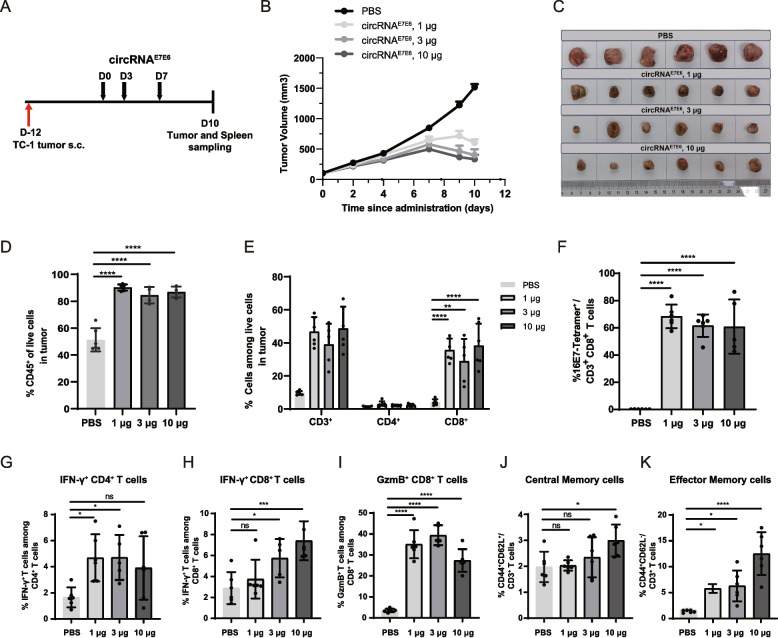
Tumor-infiltrating lymphocytes induced by circRNA ^E7E6^ vaccination. **A** Schematic diagram of the vaccination. **B** Tumor growth was monitored three times a week. **C** Representative images of tumors excised on day 10. In tumor tissues, the percentages of CD45^+^ live cells (**D**), CD8^+^ live cells (**E**), HPV16 E7 tetramer-specific CD8^+^ T cells (**F**), IFN-γ-secreting CD4^+^ T cells (**G**), IFN-γ-secreting CD8^+^ T cells (**H**), GzmB-secreting CD8^+^ T cells (**I**) were determined via flow cytometric analysis. In spleens, CD44^+^CD62L^+^CD3^+^ central memory T cells (**J**), and CD44^+^CD62L^−^CD3^+^ effector memory T cells (**K**) were detected via flow cytometric analysis. Data are shown as means ± SDs. Statistical significance was analyzed using one-way ANOVA with multiple comparisons between PBS control and circRNA^E7E6^ treatment groups. * *P* < 0.05; ** *P* < 0.01; *** *P* < 0.001, **** *P* < 0.0001, ns, not significant

To understand how circRNA^E7E6^ led to tumor regression, we characterized tumor-infiltrating T cells after vaccination. Mice were implanted with 2.5 × 10^5^ TC-1 cells and immunized with increasing amounts of circRNA^E7E6^ three times within 1 week after tumor volumes had reached an average of 100 mm^3^ (Fig. 4A).

Control mice exhibited progressive tumor growth; however, circRNA^E7E6^ vaccination (1–10 μg) induced a dose-dependent delay in tumor growth (Fig. 4B and C). Three days after the final vaccination, flow cytometry of tumor tissues revealed that circRNA^E7E6^ immunization augmented immune cell infiltration (Fig. 4D), particularly CD8^+^ T cells (Fig. 4E), and significantly expanded E7-specific CD8^+^ T cells within the tumor (Fig. 4F). Concurrently, we observed a modest expansion of Th1-polarized cells among the tumor-infiltrating CD4^+^ T cells (Fig. 4G). Tumor-infiltrating CD8^+^ T cells from mice immunized with circRNA^E7E6^ expressed high levels of IFN-γ and GzmB, unlike those from PBS-treated controls (Fig. 4H and I). These immunized mice further exhibited increased PD-1^+^ CD8^+^ T cells (Supplementary Fig. S5), indicating a potential for anti-PD-1/L1 combination therapy. Importantly, circRNA^E7E6^ treatment also induced a slight yet statistically significant elevation in the frequency of memory T cells in splenocytes (Fig. 4J, K and Supplementary Fig. S6), suggesting the presence of memory CD8^+^ T cells in the circulation and peripheral tissues for immune surveillance. These results indicated that circRNA^E7E6^ vaccination could enhance systemic cytotoxic T-cell responses and promote robust immune cell infiltration into the TME.

### circRNA^E7E6^ vaccination prevents TC-1 tumor development and recurrence

**Fig. 5:**
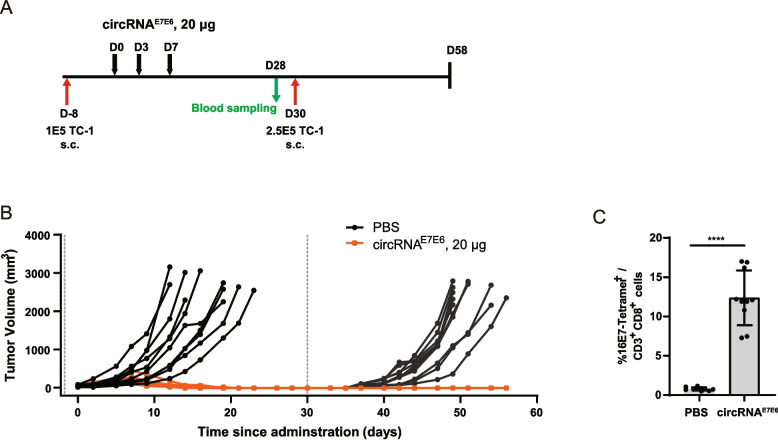
Immunization with circRNA^E7E6^ vaccine provides long-term protection from HPV16-related tumors. **A** Schematic diagram illustrating the design of therapeutic-rechallenge model. TC-1 tumor-bearing mice were treated with 20 μg of circRNA^E7E6^ on days 0, 3, and 7. Naïve mice served as controls. After the initial treatment and elimination of primary tumors, the mice were rechallenged with the same type of tumor cells in the contralateral flank on day 30. Tumor growth was monitored and measured at regular intervals. **B** Tumor growth curves. Quantification of circulating HPV16 E7 tetramer-positive CD8^+^ T cells by flow cytometry on days 28 (**C**). Data are shown as means ± SDs. Statistical significance was analyzed using an unpaired, two-tailed *t*-test between PBS control and circRNA^E7E6^ treatment groups. **** *P* < 0.0001

To assess the long-term efficacy of the circRNA^E7E6^ vaccine, we established a therapeutic-rechallenge model in C57BL/6 mice, monitoring both tumor growth and the antigen-specific CD8^+^ T cell response. C57BL/6 mice were subcutaneously inoculated with 1 × 10^5^ TC-1 cells on day-8. Once tumors reached approximately 50 mm^3^, the mice were then randomly divided into two groups and intramuscularly administered with either PBS or 20 μg of circRNA^E7E6^ vaccine on days 0, 3 and 7 (Fig. 5A). The vaccine triggered a potent anti-tumor effect, with 100% (10/10) of mice achieving complete tumor regression at day 21 (Fig. 5B). Crucially, to evaluate long-term protective immunity, these tumor-free mice were re-challenged with a secondary, high-dose TC-1 cells (2.5 × 10^5^ cells) on day 30. The vaccinated mice demonstrated effective long-term memory, remaining tumor-free until day 58, in stark contrast to the progressive tumor growth observed in age-matched naïve controls (Fig. 5B). This durable protection was underpinned by the elevated E7-specific CD8^+^ T cells detected in peripheral blood on day 28, just prior to the tumor re-challenge (Fig. 5C). These results demonstrated that the circRNA^E7E6^ vaccine established a durable, CD8^+^ T cell-mediated immune memory capable of both eradicating existing tumors and preventing subsequent recurrence.

### Immune activation-related genes upregulated after circRNA^E7E6^ treatment

**Fig. 6:**
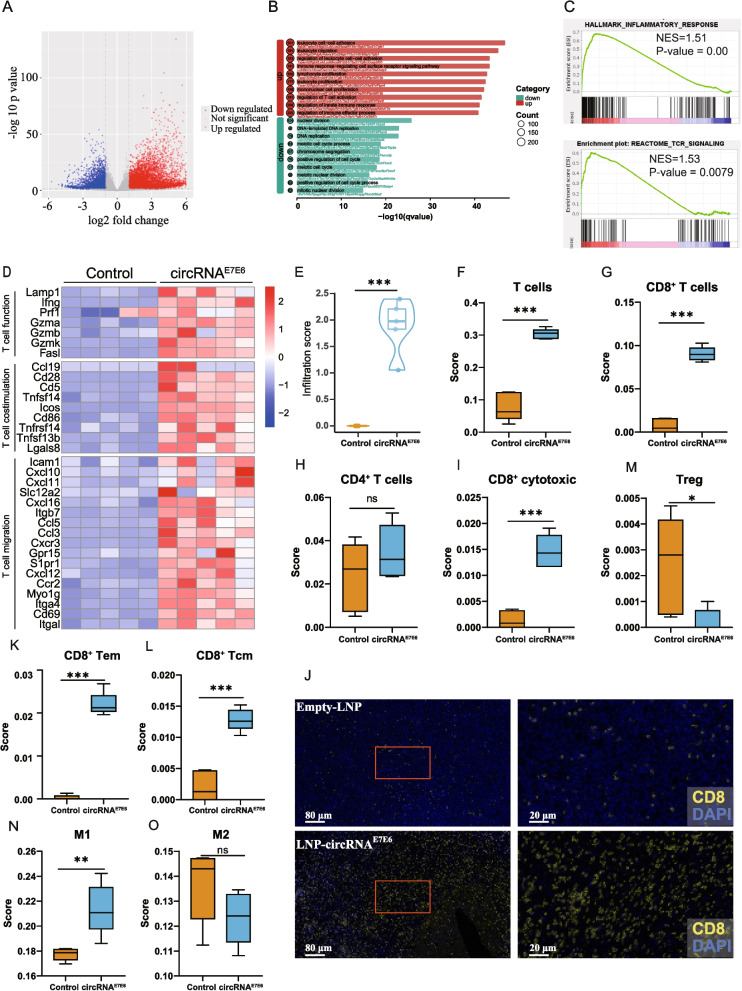
circRNA^E7E6^ vaccination elicits a robust immune activation gene signature and brisk CD8^+^ T cell infiltration. RNA-seq analysis of tumors immunized with circRNA^E7E6^ or empty LNP. **A** Volcano plots of differentially expressed genes. Differentially expressed genes were defined as those showing more than a 2-fold change in expression between control mice and circRNA^E7E6^-treated mice, with statistical significance (*p* < 0.05). **B** Gene Ontology enrichment analysis of biological processes for up- and downregulated- genes. Terms are ordered by the q value of each hit. **C** GSEA plot showing enrichment of the inflammatory response and TCR signaling in mice treated with circRNA vaccine. **D** Heatmaps showing upregulation of genes associated with T-cell function as well as T-cell activation and migration in tumors from circRNA^E7E6^-vaccinated mice. **E** Immune cell abundance estimation in tumors. Estimated immune infiltration score in the circRNA^E7E6^-vaccination group was much higher; infiltration of T cells (**F**) and CD8^+^ T cells (**G**), specifically cytotoxic (**I**), effector memory (**K**) and central memory CD8^+^ T cells (**L**), were increased. Total CD4^+^ T cells (**H**) showed an increasing trend, but regulatory T cells (**M**) were decreased. **N** Fraction of pro-inflammatory M1 was increased in circRNA^E7E6^-vaccinated mice, and M2 showed a decreasing trend (**O**). **J** Sections of tumors from mice treated with circRNA^E7E6^ or empty LNP were stained with anti-mouse CD8 antibody. Representative views of CD8 are shown

Our experimental results thus far indicated that immunization with circRNA^E7E6^ vaccine could eliminate established tumors in mice. To understand the molecular basis of circRNA^E7E6^ cancer vaccines in anti-tumor treatment, we performed RNA-seq analysis of TC-1 tumors collected 5 days after three immunizations with 2 μg of circRNA^E7E6^ vaccine or empty LNP (Supplementary Fig**.** S7A). A total of 5815 upregulated and 3036 downregulated genes were identified in the circRNA^E7E6^ treatment group, as compared with the control group treated with equivalent amounts of empty LNP (Fig. 6A). GO analysis revealed that downregulated genes were mainly associated with biological processes important for tumor cell proliferation such as nuclear division, DNA replication, and cell cycle regulation; upregulated genes were related to processes involved in leukocyte cell–cell adhesion, leukocyte migration, leukocyte proliferation, T-cell activation, and innate immune cell responses (Fig. 6B). GSEA showed that inflammatory responses and T-cell receptor (TCR) signaling pathways were activated in mice treated with circRNA^E7E6^ vaccine (Fig. 6C). All these data indicated a more active immune response in circRNA^E7E6^ vaccine-treated mice. Immunogenicity analysis in C57BL/6 mice showed robust E7-specific cytotoxic CD8^+^ T-cell responses induced by circRNA^E7E6^ vaccine. In line with that finding, gene expression analysis revealed that circRNA^E7E6^ immunization was associated with upregulation of markers for CD8^+^ T cell cytotoxic functions, as well as markers for T-cell activation and migration, such as degranulation marker CD107a (LAMP-1); T-cell costimulatory molecules CD28, ICOS, and CD86; and chemokines CXCL10 and CXCL12, together with their co-receptor CXCR3 (Fig. 6D). Gene analysis indicated significant immune cell infiltration in the TME with vaccination, which further extended the flow cytometry data on a cellular level.

### circRNA^E7E6^ treatment reshapes the tumor microenvironment (TME) to an immunostimulatory state

To further analyze the abundance of different TILs, the ImmuCellAI-mouse (Immune Cell Abundance Identifier for mouse) algorithm was used. As shown in Fig. 6E, significant immune cell infiltration was observed in tumor samples treated with circRNA^E7E6^, as compared with control samples. Consistent with the flow cytometry data (Fig. 4E), T-cell fractions, specifically cytotoxic CD8^+^ T cells, were highly increased after circRNA immunization (Fig. 6F–I). Immunofluorescence staining also verified the increase in intra-tumoral CD8^+^ T cells (Fig. 6J and Supplementary Fig. S7B and C). Of note, intra-tumoral effector memory CD8^+^ T cells (Fig. 6K) as well as central memory CD8^+^ T cells (Fig. 6L) were both significantly elevated, which mediated the long-lived anti-tumor immunity. Conversely, fractions of regulatory T cells, an immunosuppressive CD4^+^ T cell subset, decreased after circRNA^E7E6^ immunization (Fig. 6M). Additionally, the frequency of tumor-associated macrophages (TAMs) polarized toward the proinflammatory M1 subtype was significantly increased (Fig. 6N) whereas the frequency of suppressive M2 macrophages showed a decreasing trend, in comparison with control mice (Fig. 6O). Overall, circRNA^E7E6^ vaccination promoted cytotoxic and memory T-cell infiltration as well as proinflammatory TAM polarization, which reshaped the TME toward a more immunostimulatory milieu to facilitate anti-tumor immune responses.

### HPV16 circRNA^E7E6^ vaccine can be combined with PD-L1 blockade to improve antitumor responses

**Fig. 7:**
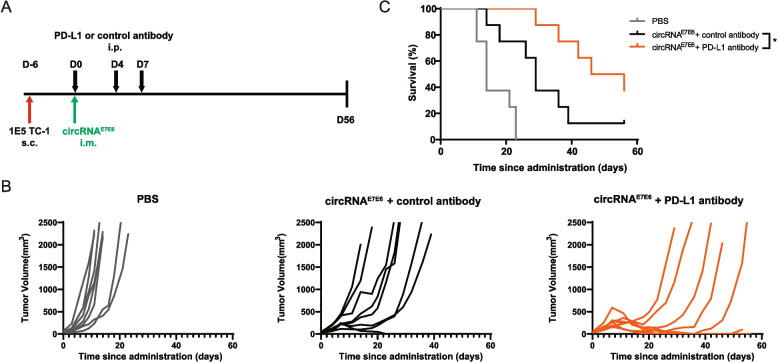
HPV16 circRNA^E7E6^ vaccine combined with PD-L1 blockade improves the antitumor response. **A** Schematic diagram of the experimental plan. **B** Tumor volume was measured and calculated according to the formula V = (L × W^2^)/2. **C** Survival curves were plotted using Kaplan–Meier survival analysis and compared by the log-rank (Mantel–Cox) test. * *P* < 0.05

The TC-1 tumor model is known to be resistant to PD-L1/PD-1 immune checkpoint blockade monotherapy [30–32], consistent with our internal evaluation (Supplementary Fig. S8). circRNA^E7E6^ immunization led to upregulation of tumor-infiltrating PD-1^+^ CD8^+^ T cells (Supplementary Fig. S5). We subsequently evaluated combination treatment with circRNA^E7E6^ and PD-L1 antibody against TC-1 tumors. Owing to the very good efficacy of circRNA vaccine monotherapy in this study, a suboptimal dosage of circRNA^E7E6^ vaccine (single dose of 1.0 μg per mouse) was administered, followed by 200 μg anti-PD-L1 or isotype control antibody treatment every 3–4 days (Fig. 7A). Combination treatment with circRNA^E7E6^ and PD-L1 blockade led to a more delayed tumor progression (Fig. 7B) and extended life span (Fig. 7C), in comparison with the combination involving isotype control antibody. The median survival for the circRNA^E7E6^ plus isotype control antibody was 29 days, compared to 46 days for the anti-PD-L1 combination group. This represents a statistically significant increase in median survival (*P* < 0.05, log-rank test). These results indicate the potential of combination therapy with PD-L1/PD-1 immune checkpoint inhibitors in clinical settings.

## Discussion

In this study, we designed a novel circRNA-based therapeutic vaccine encoding HPV E7/E6 antigens modified with a CRT signal sequence, P2 and P16 epitopes, and KDEL motif. This design simultaneously targeted the E7E6 antigen to the ER to facilitate MHC class I antigen presentation and included P2 and P16 epitopes for robust T-cell help. This strategy achieved an optimal balance between T helper cell provision and CD8^+^ T-cell activation, ultimately inducing greater immunogenicity in comparison with the non-engineered E7E6 antigen.

We provided evidence that the therapeutic efficacy of circRNA^E7E6^ is mediated by antigen-specific CD8^+^ T cell activation, robust local immune cell infiltration of tumors, as well as antigen-specific memory T-cell induction. Moreover, transcriptional levels of immune activation genes were increased in the tumor post circRNA^E7E6^ treatment, with GO and GSEA analyses confirming an enhanced anti-tumor T-cell response. Moreover, circRNA^E7E6^ reshaped the TME toward a more immunostimulatory state. Crucially, vaccination with circRNA^E7E6^ conferred a durable protective immune response, effectively preventing both eradicating existing tumors and preventing subsequent recurrence in rechallenge experiments. The delayed TGI and significant survival benefit observed when combining circRNA^E7E6^ with PD-L1 blockade are highly encouraging, suggesting that patients who are non-responsive to checkpoint inhibitors alone may benefit from such a combination, particularly in later lines of treatment.

Therapeutic vaccines for HPV-associated malignancies are a rapidly evolving field with many modalities, including peptides, protein, viral vector, bacterial vector, DNA, and mRNA-based vaccines [33–36]. Some of these have been proven to be partially successful, but no therapeutic HPV vaccine has yet been approved. Peptide-based vaccines often require potent adjuvants to overcome weak immunogenicity [37], live vector-based vaccines risk eliciting anti-vector immunity that compromises efficacy upon repeated dosing [38], and cell-based therapies are constrained by high manufacturing costs and scalability challenges [12]. Consequently, nucleic acid-based vaccines have garnered great interest owing to their capacity for in situ antigen expression, which facilitates endogenous presentation to the immune system. Among these, DNA vaccines are the most clinically advanced. For instance, VGX-3100 (Inovio Pharmaceuticals), a plasmid-based vaccine encoding HPV16/18 E6 and E7, demonstrated encouraging efficacy in a Phase III trial for cervical high-grade squamous intraepithelial lesions [39]. Other DNA vaccine candidates, such as VB10.16 (Nykode Therapeutics) and GX-188E (Genexine), are progressing through Phase II trials [40, 41]. As a promising cancer immunotherapy strategy, mRNA vaccines offer the advantages of more efficient cellular uptake without electroporation and no risk of genomic integration, in comparison with DNA vaccines. The clinical frontrunner, BNT-113 (BioNTech), is being evaluated for the treatment of HPV-associated cancers (NCT03418480, NCT04534205) and has shown good tolerability and promising immune and disease-control responses [42, 43].

Conventional linear mRNA vaccines require nucleotide modifications (e.g., 1-methylpseudouridine, m1ψ), along with optimized untranslated regions and polyA tails, to enhance translational efficiency and stability. Such modifications can introduce drawbacks, including increased costs, potential m1ψ-induced frameshift translation, and reduced intrinsic immunogenicity [44–46]. In this context, circRNA is emerging as a highly promising next-generation platform for cancer vaccine development. Its covalently closed-loop structure confers superior nuclease resistance and stability over linear mRNA, enabling effective translation without nucleotide modifications, making circRNA particularly promising for the development of more potent and effective cancer vaccines. Indeed, early studies have demonstrated that engineered circRNA vaccines can efficiently induce cytotoxic T lymphocytes and lead to significant tumor regression in animal models [47–49]. It is worth noting that a circRNA-based vaccine encoding an E6-E7-MITD fusion construct induced stronger antigen-specific T-cell immunity and exhibited superior therapeutic efficacy in mice, in comparison with conventional linear mRNA vaccines [50]. This result further supports the use of state-of-the-art circRNA-based vaccines as a novel strategy in cancer immunotherapy.

Although beyond the scope of the present study, given the immunological differences between murine models and human subjects, assessing the vaccine in non-human primates will be helpful toward clinical translation [51]. Further determining the ability of circRNA^E7E6^ to induce cross-reactive responses against other phylogenetically related HPV types, such as HPV18, should also be explored.

In summary, our study demonstrates the important therapeutic potential of the circRNA^E7E6^ vaccine in HPV16 + tumor-bearing mice. These promising preclinical results support the circRNA platform in the development of potent cancer immunotherapies, with clinical trials of the circRNA^E7E6^ vaccine planned for patients with HPV16-positive recurrent or metastatic cancers in the near future.

## Conclusions

The induction of HPV-specific CD8^+^ T cells and the observed anti-tumor effect in the TC-1 tumor model demonstrate that LNP-circRNA^E7E6^ vaccine, either as monotherapy or combined with PD-L1 blockade, represents a promising approach for treating HPV16-related solid tumors.

## Supplementary Information

Supplementary Material 1: Figure S1. Characterizations of LNP-circRNA vaccine. (A). Urea-PAGE denaturing gel electrophoresis of the linear and purified circular RNA. (B). The size distribution of LNP-circRNA^E7E6^ was measured using dynamic light scattering with a Zetasizer Pro (Malvern Panalytical Ltd., WR, UK). The data shown are from one of three biological replicates. Figure S2. P2P16-specific IFN-γ response in CD4^+^ T-cell-enriched splenocytes. (A) Quantification of IFN-γ-secreting spots and (B) representative ELISpot wells. Splenocytes were depleted of CD8^+^ T cells and stimulated with the P2P16 peptide pool. Data in (A) are presented as mean ± SDs. Statistical significance was determined by one-way ANOVA with multiple comparisons. * *P* < 0.05, ** *P* < 0.01, *** *P* < 0.001, ****P < 0.0001. Figure S3. Flow cytometry gating strategy (MHC-tetramer) Flow cytometry gating strategy for antigen-specific functional T cell subsets: Single cells > Viable Cells > CD45^+^ cells > CD3^+^ cells and CD8^+^ cells > CD8^+^ and MHC Tetramer^+^ cells. Figure S4. Flow cytometry gating strategy (ICS) (A) Flow cytometry gating strategy for ICS (intracellular cytokine staining) of different cytokines secreted by T cells subsets: Single cells > Viable cells and CD45^+^ cells > CD3^+^ cells > CD8^+^ and CD4^+^ > CD8^+^ and GzmB^+^ or CD8^+^ and TNF-α^+^ or CD8^+^ and IFN-γ^+^ or CD4^+^ and IFN-γ^+^ populations. (B) Representative flow cytometry plots showing the expression of GzmB, IFN-γ and TNF-α in CD8^+^ T cells from mice treated with PBS or immunized with circRNA^E7E6^. Figure S5. Percentage of tumor-infiltrating PD-1^+^ CD8^+^ T cells. Frequency of tumor-infiltrating PD-1^+^ CD8^+^ cells in tumors from mice 10 days after immunization with PBS or indicated doses of circRNA^E7E6^ vaccine. Data are shown as mean ± SDs. Each symbol represents one mouse. Figure S6. Flow cytometry gating strategy (Memory T cell subsets). Flow cytometry gating strategy for central memory T cell and effector memory T cell subsets: Single cells > Viable cells > CD3^+^ cells > CD8^+^ or CD4^+^ cells > CD44^+^ and CD62L^+^ or CD44^+^ CD62L^−^ populations Figure S7. Immunofluorescence analysis of TILs in TC-1 tumor bearing C57BL/6 mice. Scheme of immunization procedure in TC-1 tumor bearing C57BL/6 mice (A). Three randomly collected tumor samples from each empty-LNP (B) and LNP-circRNA^E7E6^ (C) treated mice were fixed with 4% paraformaldehyde. Serial sections of the tumor tissues were used for immunofluorescence (IF) analysis to detect the intra-tumoral CD8^+^ T cells on day 12 post immunization. Fluorescent channels and corresponding targets were labeled in each image. The yellow fluorescence channel represented CD8. Blue represents DAPI staining for nuclei. Figure S8. TC-1 tumor bearing mice is resistant to PD-L1 antibody monotherapy. (A) Schematic of experimental plan. Wild-type C57BL/6 mice were injected subcutaneously (s.c) with 2.5 × 10^5^ TC-1 tumor cells 7 days before grouping. When tumor volume reaches 60 mm^3^, mice were treated with PD-L1 antibody (200 μg per mouse) every 3–4 days via i.p. injection. (B) Tumor volume was measured and calculated according to the formula V = (L × W^2^)/2.

Supplementary Material 2.

## Data Availability

The datasets used and/or analyzed during the current study are available from the corresponding authors on reasonable request.

## Abbreviations

HPV: Human Papillomavirus
HR: High Risk
circRNA: Circular RNA
LNP: Lipid Nanoparticles
TME: Tumor Microenvironment
CRT: Calreticulin
MITD: Major histocompatibility complex (MHC) class I trafficking signal
ER: Endoplasmic Reticulum
TAP: Transporter Associated with Antigen Processing
ELISpot: Enzyme-linked immunosorbent spot
TILs: Tumor Infiltrating Lymphocytes
FFPE: Formalin-Fixed Paraffin-Embedded tissue
MHC: Major Histocompatibility Complex
ANOVA: Analysis of Variance
GO: Gene Ontology
GSEA: Gene Set Enrichment Analysis
TCR: T cell receptor
ICB: Immune checkpoint blockade
IRES: Internal Ribosome Entry Site
SD: Standard Deviation
ICS: Intracellular Cytokine Staining
Prf1: Perforin
GzmA: Granzyme A
GzmB: Granzyme B
GzmK: Granzyme K
Treg: Regulatory T cells
Fasl: Fas ligand
P53: Tumor protein p53
pRb: Retinoblastoma protein
LAMP: Lysosomal-associated membrane protein
CD40L: CD40 Ligand
i.m.: Intramuscular(ly)
i.p.: Intraperitoneal(ly)
s.c.: Subcutaneous(ly)
DSPC: 1,2-Distearoyl-sn-glycero-3-phosphocholine
SM-102: Heptadecan-9-yl 8-((2-hydroxyethyl)(6-oxo-6-(undecyloxy)hexyl)amino)octanoate
DMG-PEG2000: 1,2-Dimyristoyl-rac-glycero-3-methoxypolyethylene glycol-2000
