# Supplementary figures and images for: Circular RNA-based HPV16 therapeutic vaccine elicits potent and durable antitumor immunity
Source: J Exp Clin Cancer Res. 2026 Jan 7;45:33. doi: 10.1186/s13046-026-03640-7 (PMC12870021; doi:10.1186/s13046-026-03640-7)

A

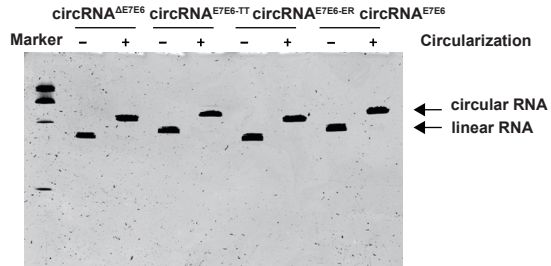

B

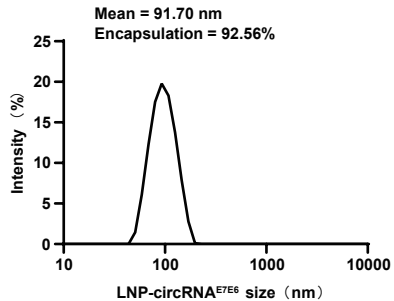

A

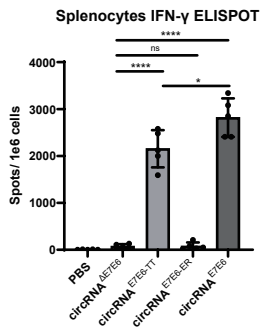

B

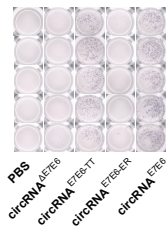

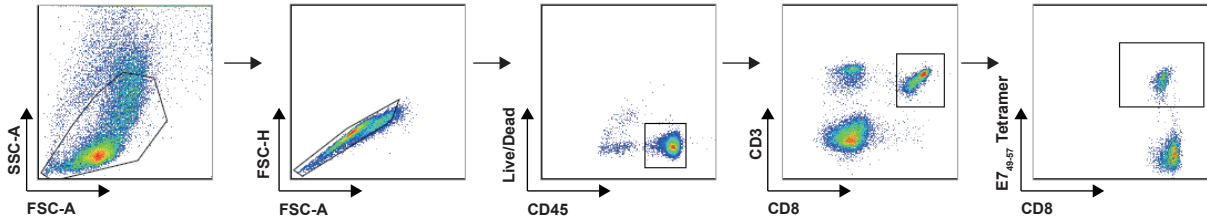

A

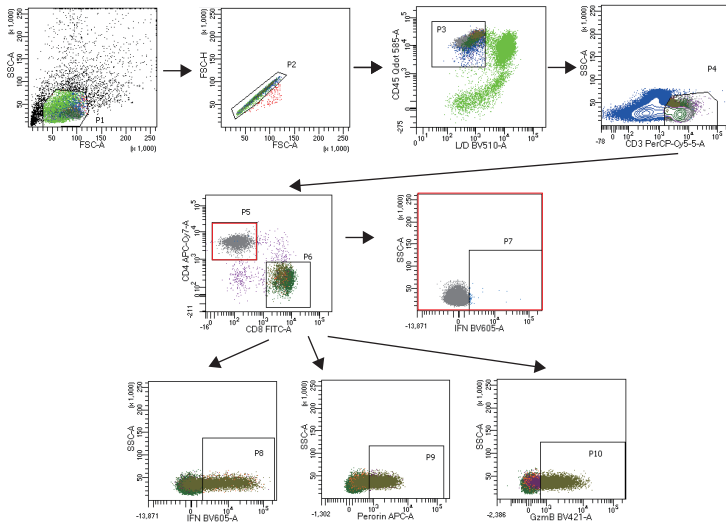

B

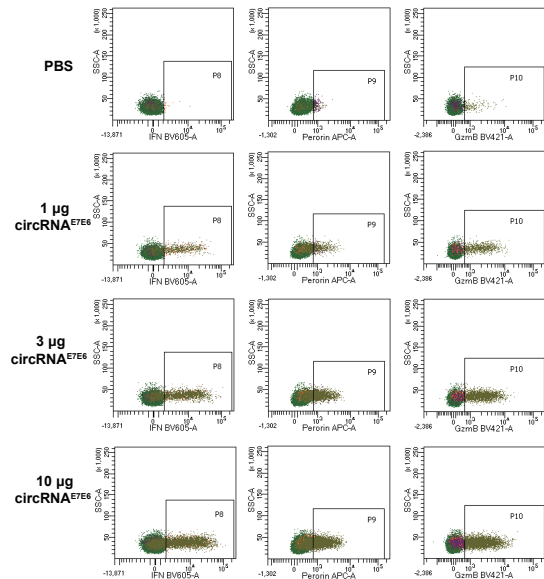

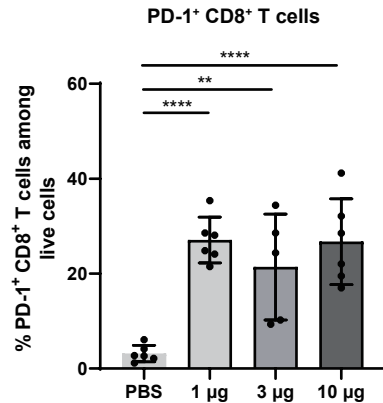

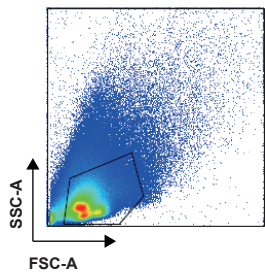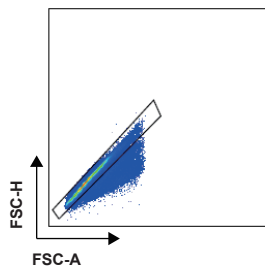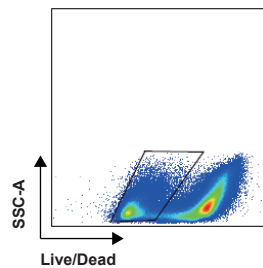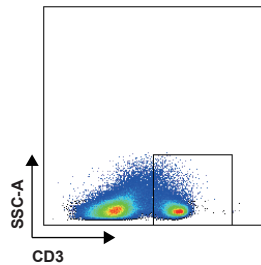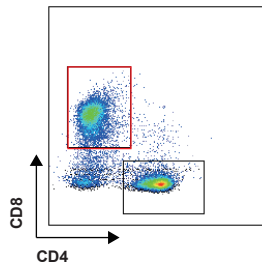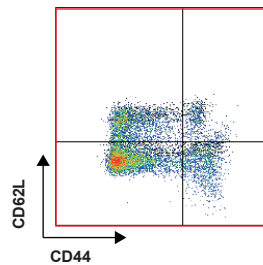

Central Memory T cells

Effector Memory T cells

A

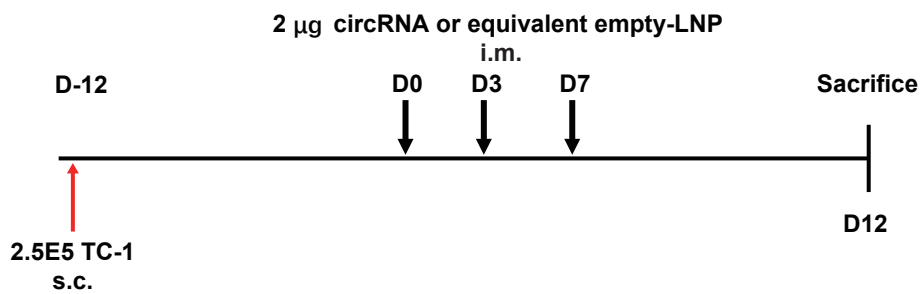

B

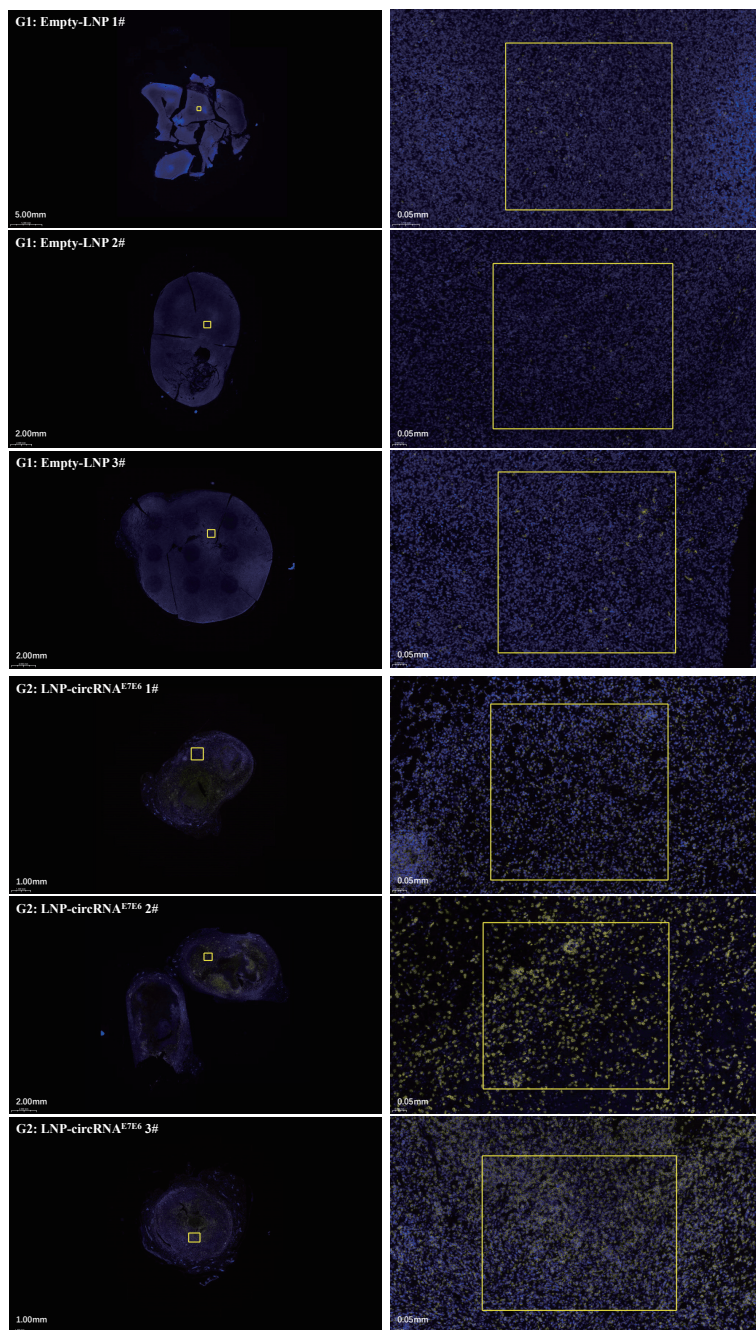

A

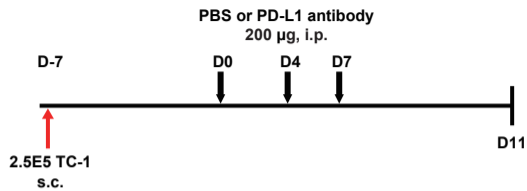

B

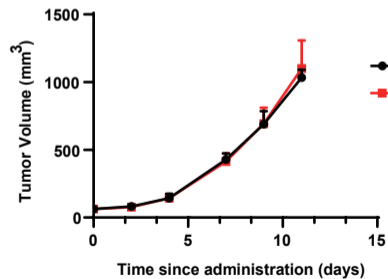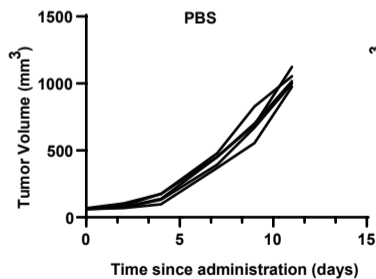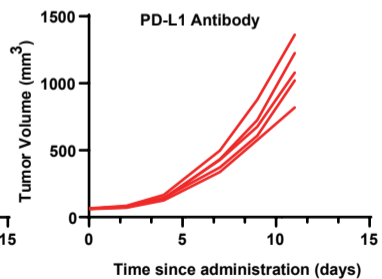

Supplement: Supplementary file 1 — Supplementary Material 1: Figure S1. Characterizations of LNP-circRNA vaccine. (A). Urea-PAGE denaturing gel electrophoresis of the linear and purified circular RNA. (B). The size distribution of LNP-circRNAE7E6 was measured using dynamic light scattering with a Zetasizer Pro (Malvern Panalytical Ltd., WR, UK). The data shown are from one of three biological replicates. Figure S2. P2P16-specific IFN-γ response in CD4+ T-cell-enriched splenocytes. (A) Quantification of IFN-γ-secreting spots and (B) representative ELISpot wells. Splenocytes were depleted of CD8+ T cells and stimulated with the P2P16 peptide pool. Data in (A) are presented as mean ± SDs. Statistical significance was determined by one-way ANOVA with multiple comparisons. * P < 0.05, ** P < 0.01, *** P < 0.001, ****P < 0.0001. Figure S3. Flow cytometry gating strategy (MHC-tetramer) Flow cytometry gating strategy for antigen-specific functional T cell subsets: Single cells > Viable Cells > CD45+ cells > CD3+ cells and CD8+ cells > CD8+ and MHC Tetramer+ cells. Figure S4. Flow cytometry gating strategy (ICS) (A) Flow cytometry gating strategy for ICS (intracellular cytokine staining) of different cytokines secreted by T cells subsets: Single cells > Viable cells and CD45+ cells > CD3+ cells > CD8+ and CD4+ > CD8+ and GzmB+ or CD8+ and TNF-α+ or CD8+ and IFN-γ+ or CD4+ and IFN-γ+ populations. (B) Representative flow cytometry plots showing the expression of GzmB, IFN-γ and TNF-α in CD8+ T cells from mice treated with PBS or immunized with circRNAE7E6. Figure S5. Percentage of tumor-infiltrating PD-1+ CD8+ T cells. Frequency of tumor-infiltrating PD-1+ CD8+ cells in tumors from mice 10 days after immunization with PBS or indicated doses of circRNAE7E6 vaccine. Data are shown as mean ± SDs. Each symbol represents one mouse. Figure S6. Flow cytometry gating strategy (Memory T cell subsets). Flow cytometry gating strategy for central memory T cell and effector memory T cell subsets: Single cells > Viable cells [file 13046_2026_3640_MOESM1_ESM.pdf]

# Uncropped gel data of figures

Fig. 1B

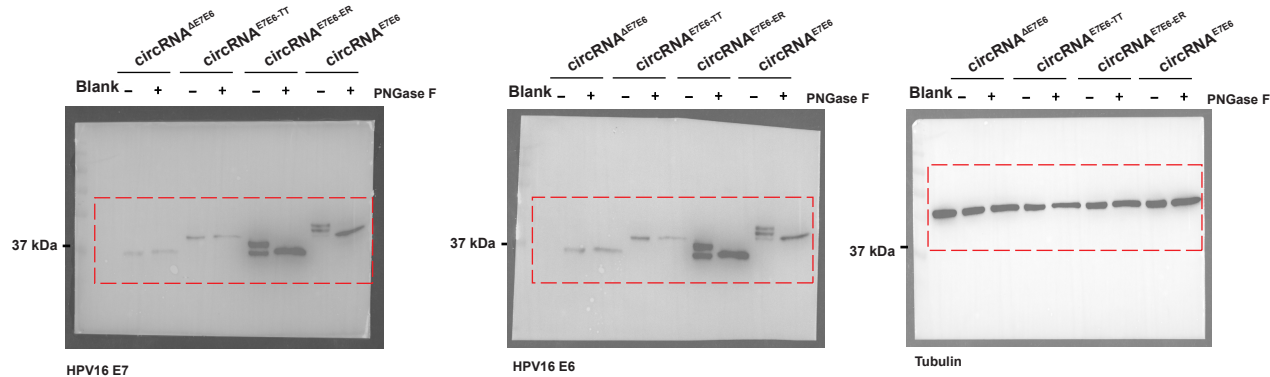

Supplementary Fig. 1A

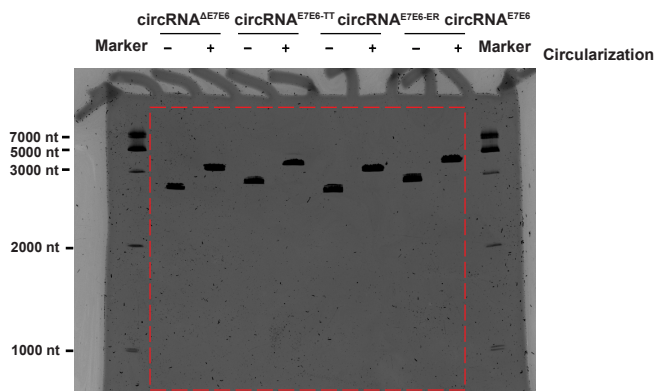

Supplement: Supplementary file 2 — Supplementary Material 2. [file 13046_2026_3640_MOESM2_ESM.pdf]
